# Hospital, Catheter, Peritoneal Dialysis Acquired Infections: Visible Light as a New Solution to Reduce Risk and Incidence

**DOI:** 10.7759/cureus.43043

**Published:** 2023-08-06

**Authors:** Joseph N Macaluso

**Affiliations:** 1 Endourology, LSU Health Foundation, New Orleans, USA; 2 Urology, LSU Health Center, New Orleans, USA

**Keywords:** hospital-acquired pneumonia, central line-associated infections (clabsi), hemodialysis complication, acute peritonitis, catheter-associated urinary tract infection, hospital, dialysis, infection, light, visible

## Abstract

Healthcare-associated infections, often identified as hospital-acquired infections (HAIs), are typically not present during patient contact or admission. Healthcare-associated infections cause longer lengths of stay, increasing costs and mortality. HAI occurring in trauma patients increases the risk for length of stay and higher inpatient costs. Many HAIs are preventable.

Antibiotic resistance has increased to a high level making proper treatment increasingly difficult due to organisms resistant to common antibiotics. Therefore, there is a need for alternate forms of attack against these pathogens. Currently, the application of light for the treatment of topical infections has been used. Ultraviolet (UV) light has well-documented antimicrobial properties. UV is damaging to DNA and causes the degradation of plastics, etc., so its use for medical purposes is limited.

Using visible light may be more promising. 405-nm light sterilization has been shown to be highly efficacious in reducing bacteria. Light Line Medical, Inc.'s (LLM) patented visible-light platform technology for infection prevention may create a global shift in the prevention of healthcare-associated infections. LLM has developed a proprietary method of delivering light to prevent catheter-associated infections. This technology uses non-UV visible light and can kill both bacteria and prevent biofilm inside and outside a luminal catheter. This is significant as prevention is key.

Independent analysis of the prototype system showed the application of the device met the acceptance criterion of 4 x 10^9-10^ reduction in Candida albicans, Staphylococcus aureus, Pseudomonas aeruginosa, and other bacteria and fungal species. Further design evolution for this technology continues, and the FDA submission process is underway.

## Introduction and background

Nosocomial infections

Healthcare-associated infections, often identified as hospital-acquired infections (HAIs), are typically not present at the time of patient contact or admission [1]. These infections usually occur after or during major healthcare interactions or hospitalization for serious illness. They become clinically evident within one or two days of contact or admission [2]. There are multiple risk factors that can predispose to these infections. Among these causes are the need for intensive care, older age, invasive procedures, intubation with ventilator use, and indwelling devices and catheters. Central vascular lines, urinary drainage devices, and ventilator use account for most occurrences within the United States [3].

In 2014, the Center for Disease Control (CDC) published a national prevalence survey of healthcare-associated infections, including over 11,000 patients from over 180 US hospitals. Per this report, 4% of inpatients suffered from at least one HAI. In 2011, CDC data showed 648,000 patients in a hospital setting suffered 721,800 infections. The dominant infections (in descending order) included Pneumonia (21.8%), surgical site infections (21.8%), gastrointestinal infections (17.1%), urinary tract infections (UTIs) (12.9%), and primary bloodstream infections (9.9%, and include Catheter-associated bloodstream infections). Among the pathogens causing HAI, Clostridioides difficile (12.1%) is the leader, followed by Staphylococcus aureus (10.7%), Klebsiella (9.9%), and Escherichia coli (9.3%) [3-5]. 

Healthcare-associated infections extend stay length, increasing cost and mortality [6]. The leading healthcare-associated infections contribute well over $9 billion in additional costs annually. Surgical site infections rank as the leading cause. Cost increases occur in every department, including the ICU [7]. Mean costs were 1.62 times higher in multidrug-resistant (MDR) bloodstream infections ($59,266 versus $36,452; P = 0.003). This persisted even after adjusting for patient factors and correct antibiotic therapy (means ratio, 1.18; 95% CI, 1.03 to 1.36; P = 0.01). Analysis of patient subpopulations indicated that increased cost of MDR BSI occurred mainly in patients with HAIs (MDR means ratio, 1.41; 95% CI, 1.10 to 1.82; P = 0.008). MDR Gram-negative bacteria are responsible for added BSI events, prolonged hospital stays, and increased costs [7]. The rise in costs is principally due to these infections [8].

Hospital, and health system incentives for investing in patient safety vary by the payor and payment configuration. Higher payments provide resources to enhance patient safety, but current payment structures may reduce the willingness of health systems to take measures to improve patient safety [8]. In most scenarios, hospitals recovered only some HAI costs through increased payments, as with Medicare and other managed care programs. Hospitals eliminated some 70% of excess costs for private patients with per-diem arrangements [9]. Costs of HAI vary between $5.7 to $6.8 billion annually [9]. 

The diagnoses with the highest annual inpatient costs (in 2006 dollars) are coronary artery disease ($17.5 billion), heart attack ($11.8 billion), and congestive heart failure ($11.2). The medical costs of preventable HAIs are comparable to the costs of major illnesses like stroke ($6.7 billion), complicated diabetes mellitus ($4.5 billion), and chronic obstructive pulmonary disease (COPD) ($4.2 billion) (Agency for Healthcare Research and Quality) [10,11].

**Figure 1:**
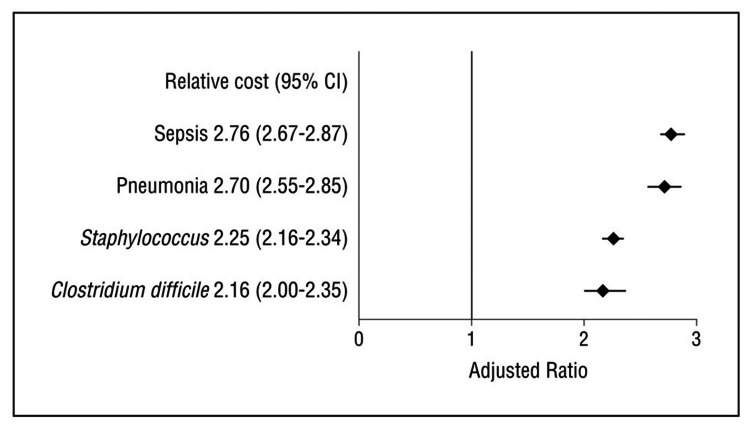
Graph of hospital-acquired infection impacts (from CDC data). Data controlled for comorbidities, injury, mechanism of injury, and demographics and adjusted for hospital factors - geographic region, urban or rural, teaching, or non-teaching, etc. CI (confidence interval).

HAI in trauma patients increases the risk for mortality, longer stay, and higher inpatient costs. Many HAIs are preventable. Given the magnitude of the clinical and economic burden of HAIs, policies to decrease HAIs have a potentially large impact on outcomes [12,13]. Also, HAI increased mortality by 1.5-2 times on average and seven times if patients became septic. Costs (Figure 1) [10] increased overall by two to threefold. Length of stay was more than doubled for any patient with HAI [12].

Some three million antibiotic-resistant infections occur every year in the United States. Thirty-five thousand plus deaths occur as a result. It is estimated that 30%-50% of all antibiotics prescribed in US acute care hospitals are unnecessary [14]. They may also often be inappropriate in selection, dose, or duration, potentially resulting in serious side effects. Adverse drug reactions and superinfections such as C. difficile infection (CDI) can occur. These events often result in major morbidity and mortality. Antibiotics save lives, yet, overusing them can lead to antibiotic resistance [15].

Drug-resistant germs called carbapenem-resistant Enterobacteriaceae, or CRE, are on the increase and have become more resistant to last-resort antibiotics during the past decade, according to a new CDC Vital Signs report [16]. These bacteria are the reason for an increasing number of infections that, in some cases, are impossible to treat. CRE are lethal bacteria that pose a triple threat: CRE are resistant to nearly all antibiotics - even our most potent last-resort drugs. Patients with CRE infections suffer high rates of mortality. 50% of patients infected by CRE die from their infection. Antibiotic resistance is readily transferred to other bacteria. Numerous examples of spread of antibiotic resistance exist, such as between CR klebsiella and normal E. coli. This then results in CR E. coli strains, a frightening scenario since E. coli is the commonest reason for UTIs in healthy people. Increasing antibiotic-resistant infections are occurring. There are numerous threats that CDC has identified. Costs due to these infections run into billions of dollars annually [17].

The Hospital-Acquired Condition Reduction Program (HACRP) links Medicare payments to quality and outcomes measures in hospitals. HACRP requires the Secretary of Health and Human Services (HHS) to regulate and adjust payments for the worst-performing 25 percent of all hospitals. Hospitals with a score > 75th percentile of HAC Scores (i.e., the worst-performing quartile) face a 1% payment reduction. Payment reductions occur when CMS pays hospital claims [17]. Scores include six quality measures [18,19]: CMS Recalibrated Patient Safety Indicator (PSI) 90 (CMS PSI 90, Centers for Disease Control and Prevention (CDC) National Healthcare Safety Network (NHSN) healthcare-associated infection (HAI) measures, Central Line-Associated Bloodstream Infection (CLABSI), Catheter-Associated Urinary Tract Infection (CAUTI), Surgical Site Infection (SSI) - colon and hysterectomy, Methicillin-resistant Staphylococcus aureus (MRSA) bacteremia, CDI [18,19].

## Review

Catheter-acquired UTI

Inpatient costs are impacted by CAUTIs due to increased tests and medications. Inpatient and outpatient hospital costs are increased by $900. This increases for non-ICU Medicare patients by over $8,300. If ICU care is needed, costs exceed $10,000 extra. In the case of pediatric patients’ costs rise by nearly $8,400 (all in 2016 $US). The common assumption that CAUTI adds ~ $1,000 to inpatient costs is an underestimate of its true economic burden. Many factors such as infection etiology, patient population, and patient acuity can increase costs well above $1,000. The cost of a CAUTI likely exceeds $1,000 [20].

Infections acquired during ICU stays are associated with a doubling of treatment costs and prolonged hospitalization. Yet, they did not significantly increase mortality [21]. Current data shows the clinical and economic impact of these infections. Infections due to MDR organisms are associated with longer hospital stays, increased costs, and higher mortality [22].

Regardless of age or sex, anyone can develop bladder infections, but women are at highest risk. Some persons are prone to these infections, especially those with lifestyle factors or medical conditions. You more easily develop a bladder infection if you do not empty your bladder completely. Conditions that may contribute to this include spinal cord injury, nerve damage around the bladder, blockage or obstruction that impedes the normal flow of urine (kidney stone or enlarged prostate), you have abnormalities of the urinary tract (vesicoureteral reflux (VUR), or UPJ obstruction), diabetes or immune system disorders, current or past use of a urinary catheter or previous UTI [23,24].

End-stage renal disease (ESRD)

ESRD is the only disease state recognized by Medicare for payment coverage no matter the age of the person. This includes all expenses related to hemodialysis (HD), peritoneal dialysis (PD), and kidney transplant (KT). Medicare expenditures for ESRD were $49.2B in 2018. Patient costs (amounts needed to be paid by patients or families) increased from almost $35 B (2009) to over $49B (2018), even adjusted for inflation.

Total Medicare FFS expenditures for ESRD increased to $36.6B in 2018, or 30.7% compared to 2009. HD, PD, and KT costs increased by 25.8%, 92.3%, and 46.2%, respectively. Rising PD expenditures were due to the increased use of PD versus HD and long waiting times for renal transplant-eligible patients. Unadjusted per patient per year (PPPY) spending on Medicare FFS ESRD beneficiaries increased from $86,939 to $93,191 for HD. PD costs rose from $67,196 to $7,874. KT patient costs increased from $33,613 to $37,304.

Inflation-adjusted PPPY expenditures for HD decreased from $102,675 in 2009 to $93,191 in 2018, or 9.2%. PPPY expenditures were minimally changed for those using PD and for those with a KT [25]. Patients undergoing dialysis treatment have a higher risk of infection, the second leading reason for death in dialysis patients.

Hemodialysis

HD filters wastes and water from blood, as kidneys did when healthy. It helps control blood pressure and balance critical minerals in the blood like potassium, sodium, and calcium. Blood goes through a filter, a dialyzer, outside the body during treatment. This dialyzer is sometimes referred to as an “artificial kidney.” At the start of treatment, the dialysis nurse or tech places two needles into the arm. Each needle is attached via a soft tube connected to the dialysis machine. Patients will usually have had a surgical procedure to place a graft in the arm or to create an arteriovenous (AV) fistula. In both, the goal is to create larger vessels into which HD catheters can be placed due to the trauma caused by the large bore needles, which are necessary [26].

PD and HD are both treatment options for end-stage renal disease in patients not eligible for kidney transplantation or it is not possible or unavailable. Over the last 20+ years, patients have preferred PD as it gives them greater freedom of activity. It is much more cost-efficient for both patients and the health system. Infection, specifically peritonitis, presents immediate health challenges and limits the ability to do PD. It often forces patients back to HD, which is more limiting, debilitating, and expensive [26,27].

Choosing PD versus HD is based on several factors. Patient desire, motivation, geographic distance from an HD unit, provider/staff bias, and patient education are all relevant. Patients are often poorly educated or not at all on PD before beginning dialysis. Risks of death with facility-based HD versus PD changes over time. PD offers lower risk, particularly in the first three months of dialysis. This advantage continues for 1.5-2 years. Beyond two years, the risk of death with PD can equal or greater than in-center HD, depending on patient comorbidities.

Thus, PD survival is best at the start of dialysis, with patient satisfaction usually higher and costs significantly lower than HD. Newer global reimbursement systems, which may include bundling of dialysis services, may lead to increasing numbers of patients on PD. Technique failures with PD persist despite reductions in peritonitis rates. Infection is also a significant cause of mortality and morbidity among HD patients. This is especially true for those using a central venous catheter as HD access. Efforts should focus on educating providers, staff, and patients about the opportunities for home therapy. A goal should be reducing reliance on central venous catheters for long-term HD access [26].

Peritoneal dialysis

PD treats kidney failure by using the lining of the abdomen (peritoneum) to filter waste from the blood. Prior to the start of PD, a unique soft tube, or PD catheter, is placed into the abdomen via a minor surgical procedure. Dialysis solution - a mixture of water, salts, and other minerals - flows from a bag through the catheter into the abdomen. Once empty, the bag is disconnected, a cap is placed on the catheter, and the patient is allowed to move around and do normal activities. The dialysis solution absorbs wastes and extra fluid from the body (dialyzing the blood of impurities).

The two forms of PD are continuous ambulatory PD (CAPD) and automated PD. Patients can choose which best fits their current lifestyle. The difference between the two is the schedule of fluid exchanges. One uses a machine, while the other is done by hand. PD complications may include infection, hernia, and weight gain. The most serious is infection. Infection around the catheter site may occur. More worrisome and of greater risk is the development of peritonitis (an infection in the fluid in the abdomen). Bacteria can enter the body through the catheter. The most significant risk is during connection or disconnection from the dialysis fluid bags. It is known that Tinkoff catheters (PD catheters) develop bacterial biofilm. Despite this, it is during active dialysis that infections are most likely to occur [27].

Site infections may cause redness, swelling or bulging, and tenderness or pain. The site may also ooze pus. Antibiotics, local and systemic, can be used to treat infections at the exit site. Peritonitis is serious. It may and often does cause pain in the abdomen, fever, nausea or vomiting, unusual color or cloudiness in used dialysis solution, redness or pain around the catheter, and cause the catheter cuff (that holds the catheter in place) to push out from the abdominal wall. Antibiotics can be added to the dialysis solution so that patients can take that home. Quick treatment is essential to prevent potentially life-threatening complications [28]. The impact of COVID-19 infections has increased the rate of HAI. Patients with a COVID-19 diagnosis who also developed an HAI saw significant increases in length of stay and costs to the health system [29].

Disinfectant technology

Many techniques are in use, almost all related to the care of medically inserted catheters (urinary, dialysis, vascular, pulmonary, etc.). This focuses on cleansing and disinfecting at the point of entry into the body and maintaining as sterile an insertion field over time as possible. However, the constant symbiosis with which all humans exist with their microbiome (native bacterial environment) means total sterility is, in fact, elusive. Thus, the medical system is on a never-ending search for newer techniques to prevent infections [30].

Studies demonstrate that application of ultraviolet (UV) light within a catheter produces a germicidal effect on microorganisms-particularly those known to be associated with peritonitis due to use of PD catheters. Yet, UV light is also known to cause degradation of many of the plastics and polymers used in catheter manufacturing [31,32]. As UV light is part of the electromagnetic spectrum is higher in energy than visible light. Higher energy x-rays and gamma rays are beyond UV on the spectrum. UV light can cause a photochemical effect within polymer structures.

Polymers of varying compositions are widely used in medical devices and tubing. That effect can be beneficial or lead to degradation of the material. Compared to human skin higher energy UV is more likely to have adverse effects on plastics. Catheter effects of UV exposure show a color change with a chalky appearance and the catheter becomes brittle and fragile. UV energy excites photons, creating free radicals as the light is absorbed into the catheter. Often pure plastics will not absorb UV radiation. Catalyst residues and impurities, acting as receptors, cause degradation within the catheter polymer makeup.

Small amounts of impurity are all that are necessary for degradation to occur. Even minute amounts (trace parts per billion of sodium) in polycarbonate begin color instability. Free radicals form oxygen hydroperoxides when exposed to oxygen. These radicals are capable of breaking the bonds that are the backbone of polymeric catheter structure. Thus, increasing fragility and brittleness in the structure, a process known as photo-oxidation. Even absent oxygen degradation will still occur. This effect is also seen in plastics used for spacecraft such as the Hubble Space Telescope and International Space Station [31,32].

Light as disinfectant

Studies have demonstrated high-efficacy bacterial reduction with the use of 405-nm light sterilization. Visible light therapy (a unique version being the PDS (Photo-Disinfection System^TM^) shows statistical significance against Gram-positive and Gram-negative species with given treatment times [33] β-lactam antibiotic-resistant E. coli is most sensitive to this PDS. This clearly shows that light therapy is suitable for sterilization in a range of drug-resistant bacterial species. Based on these findings, the potential for PDS to prevent or treat bacterial infections is significant.

The efficacy of this technology against β-lactam-resistant E. coli speaks to the need for new and innovative sterilization methods in healthcare facilities. Results suggest that PDS using 405 nm light could have clinical use for eradication of β-lactam-resistant E. coli. E. coli will be killed by visible light to a large extent. Antibiotic-resistant Gram-negative bacteria exhibit marked sensitivity to 405 nm light. Research has shown that β-lactam-resistant E. coli colonies demonstrate a >6^10^ reduction when exposed to visible light therapy. Research has increasingly demonstrated that manual cleaning and disinfection of the operating room (OR) is suboptimal. Residual environmental contamination may exist. That fact poses an infection risk to surgical wounds. Studies have been conducted on the impact of a visible-light continuous environmental disinfection (CED) system on microbial surface contamination and surgical site infections (SSI) in an OR. Recent data from studies of various surfaces in an OR demonstrate that a visible-light CED system in conjunction with manual cleaning results in significant reductions in both microbial surface contamination and SSIs (surgical site infections) in the OR [34,35].

Antibiotic resistance has dramatically increased to alarming levels. Diseases caused by resistant microorganisms to common antibiotics, even common infections, are made increasingly difficult to treat. Novel, innovative methods for inactivation of pathogens are needed. Application of light for treatment of topical infections has been used and documented. Antimicrobial properties of UV light are well known and studied. However, due to its DNA-damaging properties and the fact that it causes degradation of plastics limits its use for medical purposes [35]. Irradiation with visible light may be promising. Published research concerning inactivation of oral bacterial species utilizing visible light shows inactivation of various bacterial species, especially pigmented ones, can occur. Research continues to evaluate on the impact of visible light on biofilm [35,36]. Luedke and others have shown that the use of visible light weakens antibiotic-resistant bacteria [37,38]. Additional published studies attest to the effectiveness of visible light in disinfection and biofilm reduction [39-42].

Extensive data and publications exist on the use of visible light and related wavelengths in medical practice. There is widespread documentation of positive impacts in neonatal jaundice, seasonal affective disorder, mood disorders, sleep disorders, skin conditions and general disinfection. There are both FDA cleared, and FDA approved uses of light in medical care [43-49].

New technology 

The Light Line™ Solution

**Figure 2:**
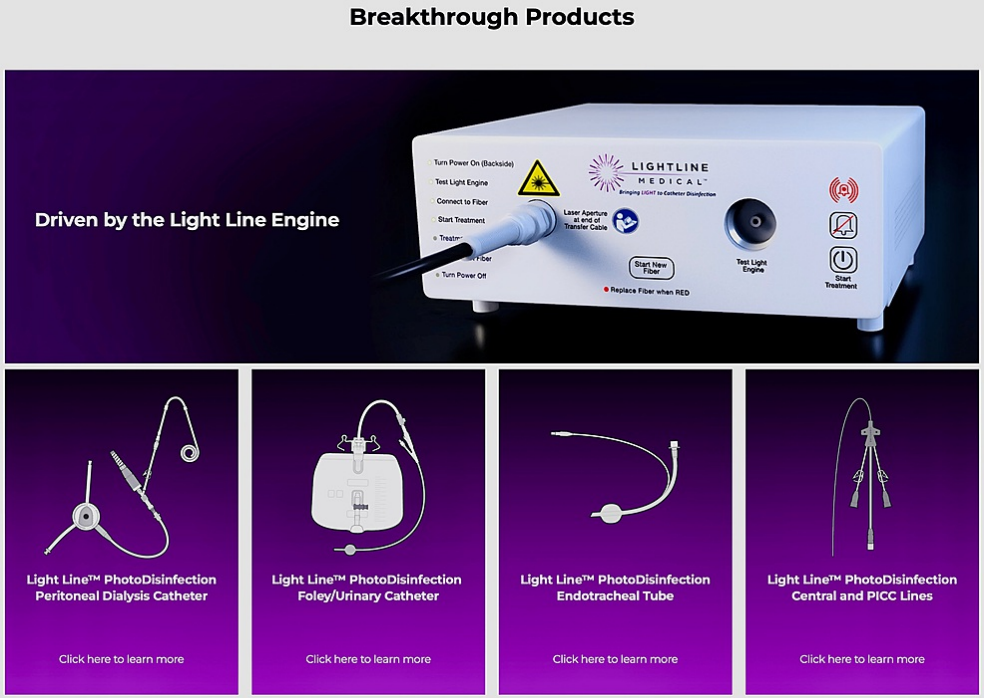
Light Line Medical interactions of product https://lightlinemedical.com/technology-products/ Used with approval Light Line Medical

CDC report that in the United States, over two million people are infected by antibiotic-resistant bacteria, and some 23,000 people die from these infections [50]. Antibiotic-resistant bacteria infections have been identified by the CDC as “one of the world's most pressing public health threats” [51]. Light Line Medical, Inc.'s (LLM) patented visible-light platform technology (Figure 2) for infection prevention may create a global shift in the prevention of healthcare-associated infections.

LLM has developed a proprietary methodology that allows light delivery to prevent catheter-associated infections. Using non-UV visible light, this technology can kill bacteria and avoid biofilm inside and outside a luminal catheter. This is significant as prevention is key. Once infection occurs with any implanted foreign body, clearing the infection is very difficult. LLM is developing four differing systems. They will all be compatible with off-the-shelf currently available catheters and endotracheal tubes. The company is not manufacturing catheters. These four products have the potential to dramatically decrease the frequency of infections that may occur related to PD, urinary tract catheters, intravascular catheters and cannulas, and endotracheal tubes for ventilators (Figure 2). Results of studies show major reductions in bacteria between control and light-treated groups. Serial electron-microscopic images from in vitro testing of LLM's prototype Foley catheter show a contrast in extra-luminal bacteria growth between test and control groups [52].

LLM's products consist of three main components that are all compatible with off-the-shelf catheters and endotracheal tubes. These include a reusable light-generating unit, a disposable fiber optic, and a fiber introducer. Studies in ovine and porcine models showed effectiveness in respiratory and UTI prevention using the LLM system [53,54].

Light Line Medical's products have three main components. In each case, they are compatible with current in-market catheters and endotracheal tubes: reusable, light-generating unit disposable fiber optic connected to the light-generating unit, which delivers light via our proprietary etching process customized fiber introducer to position and hold the fiber in place (Figure 2).

Additional applications are being evaluated. The first is the Peritoneal Dialysis Photo Disinfection System™^. ^Due to infections, 89% of ESRD patients choose HD over PD. This is despite over 65% of all dialysis patients qualifying for PD. PD is less costly and has a higher five-year survival rate. The implementation of use of the system is easily incorporated into the current multi-step training regimen taught to patients and families by nurse educators affiliated with dialysis programs.

Next is the Urinary Photo Disinfection System™ - CAUTI is among the most common infections contracted in the inpatient or long-term care setting (American Association of Critical-Care Nurses - AACCA, 2012). Urinary catheters are the typical cause. The use here adds only a minor step in application when an indwelling urinary catheter is used.

The Respiratory PhotoDisinfection System™ - Nosocomial pneumonia developing > 48-72 hours after endotracheal tube intubation (ETT) is defined as ventilator-associated pneumonia (VAP). It is the most common infection in the ICU setting. When this type of infection occurs, it is a major cause of increases in morbidity, death and costs.

Vascular Catheters PhotoDisinfection System™. One of the most serious infections is central line-associated bloodstream infection (CLABSI). It is a major cause of increased hospital stays, mortality, and cost. Typically, each year in the US there are over 30,000 incidents of CLABSI. An overview of the peritoneal system is shown in Video 1. It has refined the light delivery with precision, allowing disinfection of both the inside and outside of translucent off-the-shelf catheters. This platform technology is designed to prevent infections in dialysis, urinary, respiratory, and vascular catheters. Catheters are necessary life saving devices -- infections limit their use. With Light Line’s technology designed to prevent and treat catheter infections, patients can receive the best catheter care available.

**Video 1:** A proprietary, patented PhotoDisinfection System that uses visible light (not harmful UV light) to prevent and treat catheter-associated infections, including those caused by antibiotic resistant pathogens

Initial data

Independent testing used the sponsor-provided Peritoneal Dialysis PhotoDisinfection System disinfection system and outlined procedure. Three extension set device replicates were used. Results showed the acceptance criterion of 4 x 10^9-10^ reduction when tested against Candida albicans and a 35-minute exposure time using the 2" x 2" etch fiber. 

**Figure 3:**
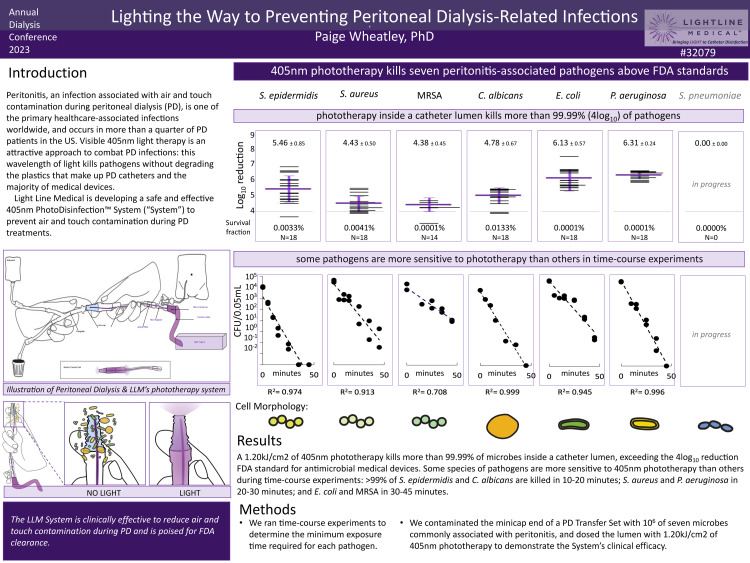
Light for preventing peritoneal dialysis-related infections Paige Wheaatley, PhD; Annual Dialysis Conference 2023, Columbia, MO Used with approval Light Line Medical

The Sponsor provided Peritoneal Dialysis PhotoDisinfection System disinfection procedure, applied to the nine extension set device replicates, meets the acceptance criterion of 4 x 10^9-10^ reduction when tested against Staphylococcus aureus and a 35-minute exposure time using the 2" x 2" etch fiber. In the opinion of the Study Director, “there were no circumstances that may have adversely affected the quality or integrity of the data” [55]. The most recent data examining the most common infectious organisms seen in PD patients and responsible for infection and peritonitis is shown in Figure 3 [56].

Based on the widespread use of visible light in medical applications, as well as the natural presence of light in daily life, there is no evidence to suggest that light exposure increases antimicrobial resistance if used with this new technology. Additionally, as outlined in the introduction, any major reduction in never events (HAI or nosocomial infections) translates into improved clinical outcomes, reduced complications and risk of mortality, as well as improved financial outcomes as increasingly care costs are fixed or pegged to set amounts. 

## Conclusions

Increasingly the status of in-hospital patient populations has grown more acute. A large percentage of patients are now treated in the office, clinic, or outpatient setting. Elective diagnostic, surgical, and interventional procedures are now performed on a same-day or overnight stay basis. The result is that many patients who remain hospitalized for any length of time are sicker and more prone to secondary infections. Measures to prevent infection rely mainly on hand washing and sterile technique in patient and staff interactions. However, despite those efforts, all intracavitary catheters (abdominal, urinary, respiratory, and vascular) are pathways for nosocomial infections. This is particularly worrisome in light of highly pathogenic bacteria, yeast, and fungi, which are increasingly resistant to medical therapies. Visible light-based technology offers an advance in the area of prevention of these costly and potentially fatal infections. Additional studies are continuing both in the laboratory and clinical settings.
